# Optimization of Storage Temperature for Cultured ARPE-19 Cells

**DOI:** 10.1155/2013/216359

**Published:** 2013-10-22

**Authors:** Lara Pasovic, Tor Paaske Utheim, Rima Maria, Torstein Lyberg, Edward B. Messelt, Peder Aabel, Dong Feng Chen, Xiangjun Chen, Jon Roger Eidet

**Affiliations:** ^1^Department of Medical Biochemistry, Oslo University Hospital, Kirkeveien 166, P.O. Box 4956, Nydalen, 0424 Oslo, Norway; ^2^Department of Ophthalmology, Oslo University Hospital, Kirkeveien 166, P.O. Box 4956, Nydalen, 0424 Oslo, Norway; ^3^SynsLaser Kirurgi Oslo/Tromsø, Lille Grensen 7, 0159 Oslo, Norway; ^4^Department of Oral Biology, Faculty of Dentistry, University of Oslo, Sognsvannsveien 10, P.O. Box 1052, Blindern, 0316 Oslo, Norway; ^5^Division of Surgery, Ear, Nose and Throat Department, Akershus University Hospital, Sykehusveien 25, 1478 Lørenskog, Norway; ^6^Schepens Eye Research Institute, Department of Ophthalmology, Harvard Medical School, 20 Staniford Street, Boston, MA 02114, USA

## Abstract

*Purpose*. The establishment of future retinal pigment epithelium (RPE) replacement therapy is partly dependent on the availability of tissue-engineered RPE cells, which may be enhanced by the development of suitable storage methods for RPE. This study investigates the effect of different storage temperatures on the viability, morphology, and phenotype of cultured RPE. *Methods*. ARPE-19 cells were cultured under standard conditions and stored in HEPES-buffered MEM at nine temperatures (4°C, 8°C, 12°C, 16°C, 20°C, 24°C, 28°C, 32°C, and 37°C) for seven days. Viability and phenotype were assessed by a microplate fluorometer and epifluorescence microscopy, while morphology was analyzed by scanning electron microscopy. *Results*. The percentage of viable cells preserved after storage was highest in the 16°C group (48.7% ± 9.8%; *P* < 0.01 compared to 4°C, 8°C, and 24°C–37°C; *P* < 0.05 compared to 12°C). Ultrastructure was best preserved at 12°C, 16°C, and 20°C. Expression of actin, ZO-1, PCNA, caspase-3, and RPE65 was maintained after storage at 16°C compared to control cells that were not stored. *Conclusion*. Out of nine temperatures tested between 4°C and 37°C, storage at 12°C, 16°C, and 20°C was optimal for maintenance of RPE cell viability, morphology, and phenotype. The preservation of RPE cells is critically dependent on storage temperature.

## 1. Introduction 

Dysfunction and loss of retinal pigment epithelium (RPE) are major pathological changes in retinal degenerative diseases such as age-related macular degeneration (AMD) and Stargardt disease. RPE cells have been shown to be good candidates for cell replacement therapy for these diseases [1–7]. With the demonstration of long-term survival of RPE cell transplants both in various animal models and in humans [8–13], transplantation offers the prospect of a single intervention cure. The transplantation of RPE grafts enables appropriate implantation and orientation of an organized RPE cell layer in the retina [5, 14, 15] and circumvents several of the complications associated with the use of RPE cell suspensions [6, 8, 15, 16]. 

In corneal transplantation, the development of storage techniques has simplified surgery logistics, enabled quality control and tissue transportation, and provided worldwide tissue availability. With the advancement of RPE cell replacement therapy, and with 20–25 million known sufferers from AMD worldwide [17], a great need for improved storage methods for cultured RPE is likely to emerge. Due to strict regulatory demands [18, 19], the development of a suitable storage method will be essential to enable the transportation of viable cell constructs from centralized laboratories to operating theatres [18]. A short-term storage method would be sufficient for this purpose, but no such protocol is available, and the optimal temperature for the short-term storage of RPE cells has not been established. 

Based on previous publications on storage of cultured epithelial cells [20–23], we hypothesize that differences in storage temperature between 4°C and 37°C affect the viability, morphology, and phenotype of cultured RPE cells. In the current study, we have used the spontaneously immortalized ARPE-19 cell line as a model. Though widely used and appreciated for displaying significant functional differentiation [24, 25], this cell line does not mirror all the functions and characteristics of native RPE [26, 27]. To ensure that the cells used in our study differ as little from primary RPE as possible, we assessed their cytoskeletal, junctional, and differential properties. 

## 2. Materials and Methods

### 2.1. Cell Culture Media and Reagents

 Cells from the adult RPE cell line ARPE-19 were obtained from the American Type Culture Collection (ATCC) (Manassas, VA). Dulbecco's Modified Eagle's Medium (DMEM): Nutrient Mixture F12, fetal bovine serum (FBS), bovine serum albumin (BSA), trypsin-EDTA, 4-(2-hydroxyethyl)-1-piperazineethanesulfonic acid (HEPES), sodium bicarbonate, gentamycin, phosphate-buffered saline (PBS), Triton X-100, penicillin, streptomycin, and 4′,6-diamidino-2-phenylindole (DAPI) was provided by Sigma-Aldrich (St. Louis, MO). Nunclon Δ-surface multidishes, glass coverslips, pipettes, and other routine plastics were supplied by VWR International (West Chester, PA). The minimum essential medium (MEM), calcein-acetoxymethyl ester (CAM), Alexa Fluor 568 phalloidin, and the primary mouse anti-ZO-1 antibody were purchased from Life Technologies (Carlsbad, CA). Staurosporine and the primary rabbit anti-cleaved caspase-3 (Asp 175) antibody were obtained from Cell Signaling Technology (Danvers, MA). The primary mouse anti-RPE65 antibody and the secondary antibodies FITC conjugated to goat anti-mouse IgG and Cy3 conjugated to goat anti-rabbit IgG were all purchased from Abcam (Cambridge, UK), while the mouse anti-PCNA antibody was obtained from DAKO (Glostrup, Denmark).

### 2.2. Cell Culture

Adult human retinal pigment epithelial (ARPE-19) cells were routinely cultured in 95% air and 5% CO_2_ at 37°C in DMEM/F12 medium containing 10% FBS, 50 units/mL penicillin,and 50 *μ*g/mL streptomycin. The cells were seeded (5000 cells/cm^2^) on Nunclon Δ-surface multidishes and glass coverslips. The culture medium was changed on the second day, and confluent cultures were obtained on the third day. Control cultures, which were not subjected to subsequent storage, were then immediately processed for the various analyses.

### 2.3. Cell Storage and Equipment

After the three-day culture period, the multidishes were removed from the incubator, and the culture medium was replaced by storage medium consisting of 1 mL MEM, 25 mM HEPES, 22.3 mM sodium bicarbonate, and 50 *μ*g/mL gentamycin. Thereafter, the cultures were randomized for storage at nine temperatures (4°C, 8°C, 12°C, 16°C, 20°C, 24°C, 28°C, 32°C, and 37°C) for seven days in custom-built storage containers without CO_2_ supply. 

**Figure 1:**
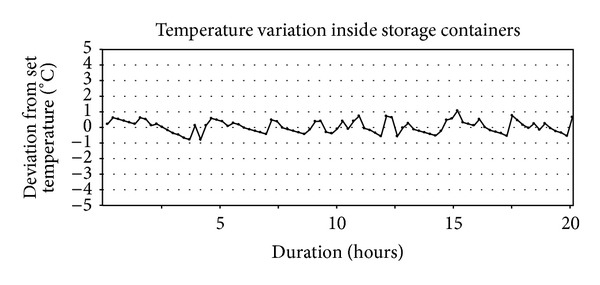
In a pilot study, the temperature inside seven of the storage containers was noted at 86 consecutive time points throughout 20 hours to assess the magnitude of the variation of the set temperature. The maximum deviation was −0.8 to +1.0°C.

 The storage containers were made from polystyrene and were kept in a cold room which maintained an ambient temperature below 4°C. All containers were equipped with (1) an electronic temperature display that enabled control of the storage temperature inside each box; (2) a heater that increased the temperature inside the box from the ambient room temperature (<4°C) to the desired storage temperature; (3) a highly sensitive thermometer that continuously regulated the heater; and (4) a small fan that ensured a homogeneous temperature inside the box by circulating the air. The stability of the temperature inside the storage containers was confirmed in a pilot study (Figure 1). In addition, the temperature inside each storage container was checked regularly throughout all experiments.

### 2.4. Viability Assessment

**Figure 2:**
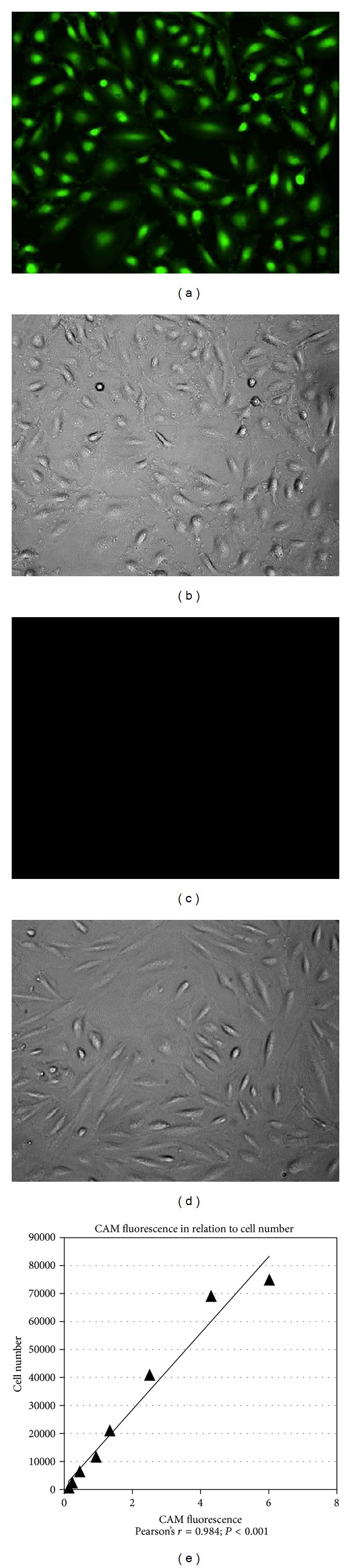
A calcein-acetoxymethyl ester (CAM) reagent, which exclusively stains living cells, was used to analyze cell survival. To validate the method, control cells and methanol-fixed cells were incubated with PBS containing 1 *μ*M CAM (green). (a) Control cells were CAM+ (green). (b) Corresponding phase contrast micrograph to (a). (c) Fixed cells were CAM−. (d) Corresponding phase contrast micrograph to (c). (e) Cells were seeded in multidishes in increasing concentrations and incubated for two hours to ensure attachment to the substrate. The CAM reagent was added to the cells for one hour, and the CAM fluorescence was measured with a microplate fluorometer. The number of seeded cells correlated significantly with the measured CAM fluorescence, thereby proving great accuracy of the microplate reader measurements.

 Viability after one week of storage was analyzed using CAM, which is enzymatically cleaved into the green fluorescent calcein inside living cells (Figure 2(a)) [28]. The cells were incubated for one hour in PBS containing 1 *μ*M CAM, and the CAM fluorescence was measured by a microplate fluorometer (Fluoroskan Ascent, Thermo Scientific, Waltham, MA) with the excitation/emission filter pair 485 nm/538 nm (*N* = 6 (repeated twice, 3 each) for 4°C, 8°C and 24°C–37°C; and *N* = 12 (repeated four times, 3 each) for 12°C–20°C). Three-day cultured cells that were not subjected to storage, but instead immediately analyzed with CAM, served as controls.

To determine the reliability of the CAM measurements obtained by the microplate reader, a standard curve was made. Using a cell counter (Scepter 2.0 Cell Counter, Merck Millipore; Billerica, MA), cell suspensions with increasing cell concentrations were seeded in multidishes and left for two hours to ensure cell attachment. The cells were then incubated with the CAM reagent as described above to stain the attached cells. The CAM fluorescence was thereafter measured by the microplate reader. The number of seeded cells correlated highly with the measured CAM fluorescence, thereby showing great accuracy of the microplate reader measurements (Pearson's *r* = 0.984; *P* < 0.001) (Figure 2(e)). 

### 2.5. Morphology Analysis

ARPE-19 cells were cultured on glass coverslips, and the samples were stored at nine temperatures for seven days before being processed for scanning electron microscopy (SEM) as previously described (*N* = 8 (repeated twice, 4 each) for 4°C, 8°C, and 24°C–37°C; *N* = 12 (repeated three times, 4 each) for 12°C–20°C) [29]. In brief, stored cultures were fixed in 2.5% glutaraldehyde solution, dehydrated in ethanol, and dried in compliance with the critical point method (Polaron E3100 Critical Point Drier; Polaron Equipment Ltd., Watford, UK). The control cultures were processed for SEM without delay after the three-day culture period. Coating of the samples with a 30 nm thick layer of platinum in a Polaron E5100 sputter coater was done prior to photographing with an XL30 ESEM electron microscope (Philips, Amsterdam, The Netherlands). 

### 2.6. Phenotype Analysis

Cells were cultured in 24-well multidishes and stored at 12°C, 16°C, and 20°C as described above. Samples were subsequently prepared for immunocytochemical characterization by 15 minutes of methanol fixation at room temperature followed by 30 minutes of permeabilization and blocking in PBS containing 1% BSA and 0.2% Triton X-100. Control cells were processed for immunocytochemistry immediately after the three-day culture period.

**Figure 3:**
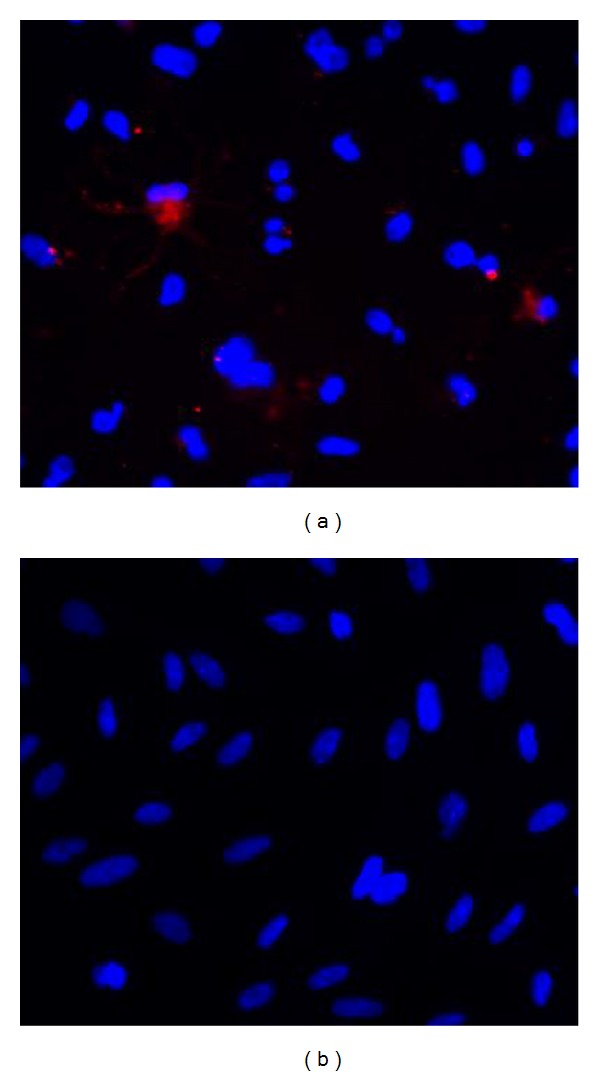
Positive control to the caspase-3 antibody. Cultured RPE cells were incubated with 1 *μ*M staurosporine for 24 hours in order to trigger expression of caspase-3 and induce cell apoptosis. (a) Photomicrograph showing immunostaining of caspase-3 (red) in apoptotic cells treated with staurosporine. Nuclei were stained with DAPI (blue). Original magnification: ×200. (b) Negative control showing only DAPI staining (blue) when no staurosporine is added. Original magnification: ×200.

Anti-ZO-1 (1 : 50), anti-RPE65 (1 : 200), anti-PCNA (1 : 1000), and anti-cleaved caspase-3 (1 : 400) antibodies were diluted in blocking solution (PBS with 1% BSA). Primary antibodies were omitted from the negative controls. Samples were incubated overnight at 4°C. Goat anti-mouse FITC-conjugated secondary antibodies (diluted 1 : 250 in blocking solution) and goat anti-rabbit Cy3-conjugated secondary antibodies (diluted 1 : 10000 in blocking solution) were added for one hour at room temperature. Specimens were washed three times in PBS, with the addition of 1 *μ*g/mL DAPI during the last wash to stain the cell nuclei. Positive control cultures for caspase-3 included incubating cells with 1 *μ*M staurosporine for 24 hours (Figure 3). Treatment with staurosporine is expected to trigger expression of caspase-3 and induce cell apoptosis [30]. 

To visualize the actin cytoskeleton, samples were fixed in 4% formaldehyde for 10 minutes, permeabilized with PBS containing 0.1% Triton-X, and stained with PBS containing 25 *μ*L/mL Alexa Fluor 568 phalloidin methanolic stock solution. After incubating for 20 minutes in room temperature, specimens were washed in PBS and stained with DAPI. 

The specimens were studied using a Nikon Eclipse Ti fluorescence microscope and photographed at ×200 magnification with a DS-Qi1 black-and-white camera. Photomicrographs were captured at five predetermined positions in each culture using a motorized microscope stage. The exposure length and gain was maintained at a constant level for all samples, and the image brightness was within the dynamic range of the camera. Two blinded and independent investigators assessed expression of the various markers in five photomicrographs in each culture (*N* = 8 (repeated twice, 4 each)). For the RPE65, PCNA, and caspase-3 markers, the number of positive cells/total number of cells × 100% was calculated. Assessment of observer agreement between the two investigators demonstrated high reliability of the phenotypic data (Table 1).

### 2.7. Statistical Analysis

A one-way analysis of variance with Tukey's post hoc comparisons (SPSS ver. 19.0) was used for statistical evaluation of the results from the viability and phenotype analyses. Pearson's correlation and a paired sample Student's *t*-test were utilized to calculate observer agreement of the phenotype data. *P* values below 0.05 were considered significant. 

## 3. Results

### 3.1. Viability of Cultured ARPE-19 Cells following Storage

**Figure 4:**
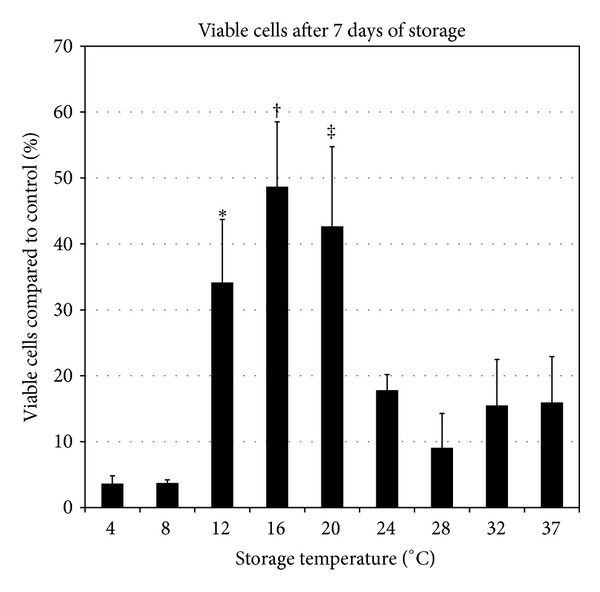
Cultured RPE cells were stored for seven days at 4°C, 8°C, 12°C, 16°C, 20°C, 24°C, 28°C, 32°C, and 37°C, and viability was assessed with a calcein-acetoxymethyl ester reagent. The bar chart shows the percentage of viable cells after storage compared to control cells (100%). *P < 0.01 compared to 4°C, 8°C, 28°C, and 37°C; *P* < 0.05 compared to 24°C and 32°C. ^†^ *P* < 0.01 compared to 4°C, 8°C, and 24°C–37°C; *P* < 0.05 compared to 12°C. ^‡^ *P* < 0.01 compared to 4°C, 8°C, and 24°C–37°C. Error bars represent the standard deviation of mean values.

To study the impact of different temperatures on RPE cell survival, cell viability was analyzed using CAM. Sealed multidishes with ARPE-19 cell cultures were randomized for storage at 4°C, 8°C, 12°C, 16°C, 20°C, 24°C, 28°C, 32°C, and 37°C for seven days. The number of live cells after seven days of storage, as indicated by the CAM fluorescence measurements, was reduced at all storage temperatures compared to the control (Figure 4). Storage at 16°C conserved the highest number of live cells (48.7% ± 9.8%; *P* < 0.01 compared to 4°C, 8°C, and 24°C–37°C; *P* < 0.05 compared to 12°C). Twenty degrees storage conserved 42.7% ± 12.1% of live cells (*P* < 0.01 compared to 4°C, 8°C, and 24°C–37°C), while storage at 12°C conserved 34.2% ± 9.6% of viable cells (*P* < 0.01 compared to 4°C, 8°C, 28°C, and 37°C; *P* < 0.05 compared to 24°C and 32°C). Thus, the temperatures 16°C and 20°C were superior for cell survival.

### 3.2. Morphology of Cultured ARPE-19 Cells following Storage

**Figure 5:**
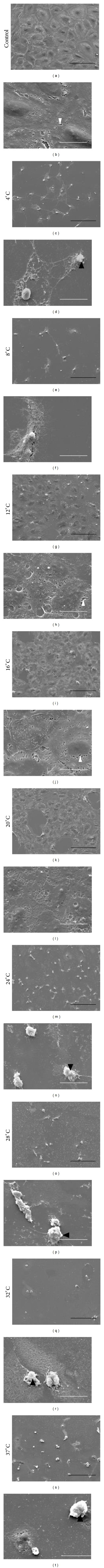
Photomicrographs of control cells and cells stored for seven days at 4°C, 8°C, 12°C, 16°C, 20°C, 24°C, 28°C, 32°C, and 37°C were captured by a scanning electron microscope. An epithelial cobblestone morphology can be seen in the control ((a), (b)), and this was best maintained after storage at 12°C, 16°C, and 20°C ((g)–(l)). The cells demonstrate apoptotic morphological alterations like shrinkage and membrane blebbing after storage at temperatures below 12°C ((c)–(f)) and above 20°C ((m)–(t)). Images are representative of three independent samples. Scale bars: 100 *μ*m (black), 20 *μ*m (white). Black arrowheads: shrinkage and membrane blebbing. White arrowheads: microcracks representing artifacts due to sample preparation.

**Figure 6:**
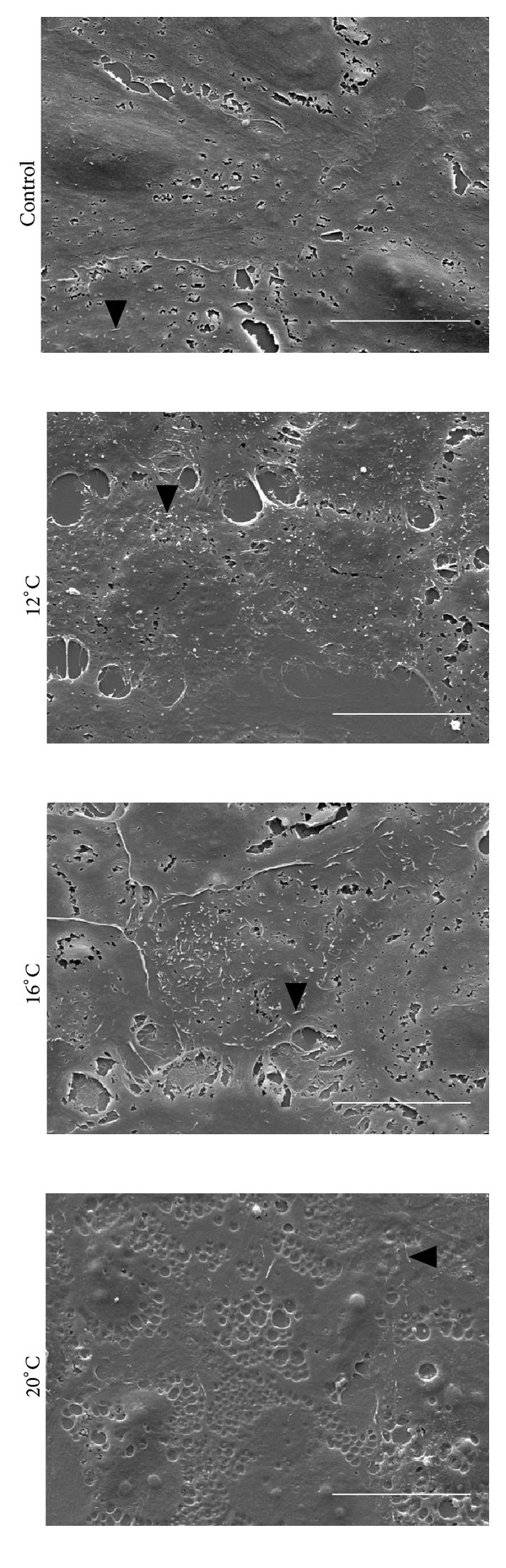
Scanning electron photomicrographs showing apical microvilli on control ARPE-19 cells as well as on cells subjected to seven days of storage at 12°C, 16°C, and 20°C. Scale bars: 20 *μ*m. Black arrowheads: microvillus.

Scanning electron microscopy was performed to investigate the effect of storage temperature on the ultrastructure of cultured RPE cells. Prior to storage, the cells were generally well apposed and displayed an epithelial morphology (Figures 5(a)-5(b)). After storage, the ultrastructure was best maintained in the 12°C, 16°C and 20°C, groups (Figures 5(g)–5(l)). Cell-cell contact was mostly preserved at these three temperatures, although some intercellular spacing was seen. There were only occasional cells with apoptotic morphology (Figures 5(g)–5(l)). After storage at temperatures below 12°C and above 20°C, on the other hand, the majority of the remaining cells showed signs of cell damage and apoptosis. These signs included extensive loss of cell-cell contact, cell detachment, shrinkage, and membrane blebbing (Figures 5(c)–5(f) and 5(m)–5(t)). Apical microvilli were found in control cultures and cultures stored at 12°C, 16°C, and 20°C, while few to no microvilli were found in cells stored at other temperatures (Figure 6). Collectively, these results were in agreement with the viability data, showing best cell preservation at 12°C, 16°C, and 20°C.

### 3.3. Phenotype of Cultured ARPE-19 Cells following Storage

**Figure 7:**
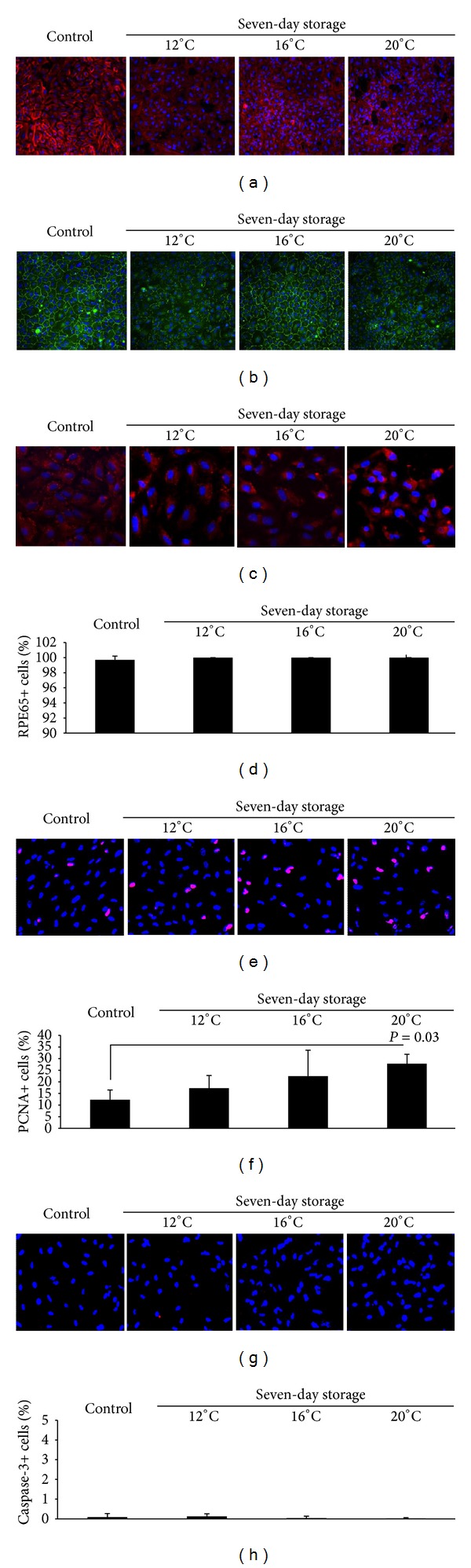
Cultured RPE cells were stored at 12°C, 16°C, and 20°C for seven days, and expression of actin, ZO-1, RPE65, PCNA, and caspase-3 was assessed. The percentage of cells expressing RPE65, PCNA, and caspase-3 was quantified by two independent and blinded investigators. (a) Photomicrographs showing immunostaining with phalloidin-Alexa 568 used to visualize actin filaments (red). Nuclei were stained with DAPI (blue). Original magnification: ×200. (b) Photomicrographs showing immunostaining of ZO-1 (green). Nuclei were stained with DAPI (blue). Original magnification: ×200. (c) Photomicrographs showing immunostaining of RPE65 (red). Nuclei were stained with DAPI (blue). Original magnification: ×200. (d) Bar chart demonstrating RPE65 expression in stored and control cells. Expression of RPE65 was maintained after storage at all three temperatures. Error bars: standard deviation of mean values. (e) Photomicrographs showing immunostaining of PCNA (red) in control and stored cells. Nuclei were stained with DAPI (blue). Original magnification: ×200. (f) Bar chart displaying the percentage of PCNA+ cells in the control cultures and in the storage groups. PCNA expression was maintained at 12°C and 16°C and increased after storage at 20°C compared to the control. Error bars: standard deviation of mean values. (g) Photomicrographs of cells stained with anti-caspase-3 antibody (red). Nuclei were stained with DAPI (blue). Original magnification: ×200. (h) Bar chart showing the percentage of caspase-3+ cells. There was no increase in caspase-3+ cells after storage compared to control. Error bars: standard deviation of mean values.

To assess the effect of storage temperature on the phenotype of cultured RPE, cells stored at 12°C, 16°C, and 20°C were immunostained with five different markers. Alexa Fluor 568 phalloidin staining was used for selective labeling of F-actin in order to visualize the cytoskeleton and assess the formation of stress fibers [27]. Actin staining revealed that the control cultures were the most heterogeneous, exhibiting actin arranged in stress fibers in some cells and circumferentially in others. Cultures stored at 12°C, 16°C, and 20°C predominantly expressed circumferential actin arrangement and fewer elongated cells than the control cultures (Figure 7(a)). 

To assess the integrity of the intercellular junctions, an anti-ZO-1 antibody was used. The antibody localized to cell borders and was present between all apposed cells. It revealed a predominance of polygonal cells in all groups, with a few elongated cells present only in the control cultures (Figure 7(b)). 

Anti-RPE65 was used to detect RPE65, a protein crucial for the regeneration of visual pigment (Figure 7(c)) [7, 31]. RPE65 expression was demonstrated in 99.7% ± 0.5% of control cells and in all cells following storage at 12°C, 16°C, and 20°C (100%; *P* = 0.52 compared to control) (Figure 7(d)). 

An anti-PCNA antibody was employed to detect proliferating cells (Figure 7(e)). The percentage of PCNA+ cells in the control was 12.3% ± 4.2% (Figure 7(f)). The expression level was maintained after storage at 12°C (17.3% ± 5.4%; *P* = 0.73) and 16°C (22.5% ±  11.1%; *P* = 0.21) and increased after storage at 20°C (27.8% ± 4.1%; *P* = 0.03).

To assess the percentage of dead cells, the cultures were immunostained with anti-caspase-3, an indicator of apoptosis (Figure 7(g)). As expected, the control cultures showed very few caspase-3+ cells (0.09% ± 0.18%) (Figure 7(h)). The percentage of caspase-3+ cells did not increase after seven-day storage at 12°C (0.12% ± 0.14%; *P* = 0.98), 16°C(0.05% ± 0.09%; *P* = 0.96), or 20°C (0.02% ± 0.04%; *P* = 0.86). These results support the morphology analyses that demonstrated only infrequent cells with apoptotic features in the 12°C, 16°C, and 20°C groups. 

## 4. Discussion

The present study shows that storage temperature has a crucial impact on the morphology and viability of cultured RPE cells. The storage temperature interval 12°C to 20°C was superior in preserving cell viability, morphology and phenotype. 

Maintaining cell viability before transplantation is critically important for optimal graft survival and function. In ophthalmology, the technique of tissue preservation is mostly utilized for storage of corneas in eye banks. Corneas are either cold-stored at 4°C or organ cultured at 31–37°C [32]. However, it is questionable whether these temperatures are optimal for maintaining RPE cell quality. In the present study we demonstrated that storage at 16°C and 20°C maintained the largest amount of live cells and moreover provided far superior results than cold conditions. The viability of adult primary RPE cell sheet grafts after storage has previously been investigated by Tezel and coworkers using a calcein and ethidium homodimer viability kit [33]. This group used cell counting rather than a microplate fluorometer. In that study, the ratio of live cells to the total cell number decreased to 32.4% after four days of storage at 4°C. In the current study, we stored the cells for seven days instead of four, and in line with the study by Tezel and coworkers, the 4°C storage group showed a great drop in the number of live CAM-retaining cells compared to the control. It should be noted that we compared the number of live cells after storage with the number of live cells in the control, whereas Tezel and coworkers compared the ratio of live cells to the total cell number in the stored cultures. However, both of these studies suggest that 4°C is not the optimal temperature for RPE cell storage. The intriguing finding of the current study, that the best storage temperature for cultured RPE cells is approximately midway between the traditional temperatures for cell culture (37°C) and cold storage (4°C), is supported by studies on other epithelial cell types. Raeder and associates reported that the storage of cultured human limbal epithelial cells at 23°C is superior to storage at both 5°C and 31°C [20], while another study reported that cultured human conjunctival epithelium maintained viability after four days of storage at 23°C in HEPES-MEM [23]. Hypothermia has been shown to reduce both ARPE-19 cell metabolism and vascular endothelial growth factor secretion in a temperature-dependent fashion [34]. We speculate that the temperature decrease to 16°C halts ARPE-19 cell metabolism to such an extent that cell survival is improved compared to higher temperatures. 

The ARPE-19 cell line used in the current study showed an epithelial morphology similar to that demonstrated previously with subcultures of this cell line [24]. Storing the cells at temperatures between 12°C and 20°C ensured the best preservation of ultrastructure, although increased intercellular spacing was seen after storage at all temperatures. Some of the intercellular gaps, however, represented microcracks due to critical point drying as part of sample preparation for scanning electron microscopy [35]. Apical microvilli, which have been demonstrated in ARPE-19 previously [31], have been reported to decrease in number in aging RPE cells [36]. The loss of apical microvilli causes unfavorable effects on the RPE cell functions and may accelerate degenerative processes in the retina [36, 37]. Hence, our results showing the preservation of microvilli only at a specific temperature range further emphasizes the need for careful temperature control during RPE cell storage. 

The actin cytoskeleton is involved in many cellular functions, affecting cell adhesion, morphogenesis, and phagocytosis [38]. Stress fibers are contractile bundles of actomyosin that are assembled when cells encounter mechanical stress [38]. Their presence *in vivo* is usually confined to muscle cells and myofibroblasts in dermal wound tissue [39], but they are common in epithelial cells cultured *in vitro* [39]. Formation of actin stress fibers in the ARPE-19 cell line has been reported earlier, and it has been demonstrated that the cells' propensity for developing these fibers depends both on culture length and the composition of the culture medium [27]. To ensure that the ARPE-19 cells used in the present study displayed normal epithelial characteristics, the actin cytoskeleton was visualized with phalloidin-Alexa 568. The staining revealed that, prior to storage, the actin filaments were mostly arranged in circumferential bands but that a subset of cells were elongated and displayed stress fiber formation. After storage at 12°C, 16°C, and 20°C, actin staining revealed no stress fiber formation, indicating both preservation and promotion of normal epithelial characteristics of ARPE-19 cells stored in serum-free HEPES-MEM. In support of this finding, Luo et al. [27] have reported a reduced tendency of stress fiber formation in ARPE-19 cells cultured in serum-free medium when compared to cells cultured in serum-supplemented medium. 

To assess the presence of intercellular tight junctions, staining with anti-ZO-1 antibody was performed. The marker localized to cell borders and was present between all apposed cells, indicating a tight junction organization typical of native RPE and revealing a cobblestone morphology with a predominance of polygonal cells in all groups (Figure 7(b)). 

Compared with the control, we did not detect a different RPE expression profile following storage at 12°C, 16°C, and 20°C. The RPE65 protein is considered an essential marker of RPE cell differentiation [31]. Even though the ATCC recommends a FBS-containing culture medium [40], storage of ARPE-19 cells in serum-free HEPES-MEM did not apparently affect the expression of the differentiation marker RPE65.

Proliferating cell nuclear antigen (PCNA) expression was maintained after storage at 12°C and 16°C but increased after storage at 20°C. Maintenance of PCNA expression during storage has also been reported for cultured human conjunctival epithelium kept for seven days at 23°C in HEPES-MEM [23]. According to Rieder and Cole, transition through the G_2_ and M phases of mitosis comes to a halt when the temperature is lowered to approximately 16–20°C, thereby prolonging the cell cycle [41]. The tendency for a progressively decreasing percentage of PCNA+ cells with lower storage temperature in our study could be related to the inhibited G_2_/M transition below 16–20°C. Upon heating, cells that have been stored at 19°C proliferate at an even higher rate than that of control cells maintained at 37°C [41]. The RPE cell layer is mitotically inactive *in vivo* [42], but has the ability to grow by cell enlargement if damage occurs [6, 42]. Both transplanted freshly harvested RPE and transplanted cultured RPE are capable of proliferating *in vivo*, but proliferation is halted upon close apposition to the neural retina, indicating an effect of the neural retina in stalling RPE cell proliferation [43]. Thus, it can be expected that the stored RPE cells will eventually stop dividing following transplantation. Some initial proliferative activity may be advantageous, as it could enable transplanted cells to cover exposed areas of Bruch's membrane [33, 44]. 

In the present study, we did not detect an increase in caspase-3+ apoptotic cells after storage. However, the number of CAM+ live cells after storage dropped to less than 50% compared to the control. The low percentage of caspase-3+ cells can be explained by the dead cells' tendency to detach and be washed away during rinsing prior to immunostaining. In support of this assumption, we found very few cells demonstrating an apoptotic morphology after storage at 12°C, 16°C, and 20°C. 

Several laboratories have investigated the cultivation of RPE cells on artificial substrates, aiming to identify carrier materials that could be directly transplanted into the subretinal space [15, 45, 46]. However, there is currently no consensus in regard to the future use of culture substrates for RPE transplantation. In the current study, ARPE-19 cells were cultured on glass or plastic culture dishes, reducing the culture variables to a minimum and allowing the impact of storage temperature on cultured cell sheets to be isolated. In support of this study design, it has been shown that differences in the ARPE-19 transcriptome can be attributed to culture conditions and that culturing of ARPE-19 cells on plastic substrates is superior in maintaining a phenotype closest to native RPE cells [47]. A storage period of seven days should be sufficient time to allow transportation from the culture laboratories to the clinics, sterility control, and preparation for the transplantation procedure. Furthermore, a major advantage of the current storage method is the ability for cell preservation without CO_2_ incubation, thereby facilitating transportation. The methods used in the current study are, however, not directly clinically applicable, and further studies on the validation of our storage technique using clinically applicable RPE cell sources and carrier substrates are warranted. Future studies aimed at identifying storage conditions and specific growth stimulating factors for RPE cell maintenance could further refine the technology to improve cell survival following storage.

## 5. Conclusion

In conclusion, this study demonstrates that human cultured RPE cells are best preserved at the temperature range 12°C to 20°C. The capability to preserve RPE cells is essential for the future advancement of RPE cell replacement therapy. Moreover, the storage method described in the current study may be applicable for other cell types and tissues; thus its significance may extend well beyond RPE and eye diseases. 

## Figures and Tables

**Table 1:** Characterization of retinal pigment epithelial cells.

Markers	Specificity	Investigator criteria for positive staining	Investigator A and B agreement	
			Significance level of correlation (*r*) between investigators	95% CI of difference between investigators
RPE65	Differentiated cells (cytosol/membrane)	Stained cytosol	*P* < 0.001	−0.1% to +0.0%
PCNA	Proliferating cells (nucleus)	Stained nucleus	*P* < 0.001	−3.3% to +0.8%
Caspase-3	Apoptotic cells (mainly cytosol)	Stained cytosol	*P* = 0.044	−0.2% to +0.0%

CI: confidence interval; *r*: Pearson's correlation coefficient.
